# Bioinformatics analysis for the identification of differentially expressed genes and related signaling pathways in *H. pylori-*CagA transfected gastric cancer cells

**DOI:** 10.7717/peerj.11203

**Published:** 2021-04-15

**Authors:** Dingyu Chen, Chao Li, Yan Zhao, Jianjiang Zhou, Qinrong Wang, Yuan Xie

**Affiliations:** Key Laboratory of Endemic and Ethnic Diseases , Ministry of Education, Guizhou Medical University, Guiyang, China

**Keywords:** Helicobacter pylori, CagA, Gastric cancer, Transcriptomics, Bioinformatics analysis

## Abstract

**Aim:**

*Helicobacter pylori* cytotoxin-associated protein A (CagA) is an important virulence factor known to induce gastric cancer development. However, the cause and the underlying molecular events of CagA induction remain unclear. Here, we applied integrated bioinformatics to identify the key genes involved in the process of CagA-induced gastric epithelial cell inflammation and can ceration to comprehend the potential molecular mechanisms involved.

**Materials and Methods:**

AGS cells were transected with pcDNA3.1 and pcDNA3.1::CagA for 24 h. The transfected cells were subjected to transcriptome sequencing to obtain the expressed genes. Differentially expressed genes (DEG) with adjusted *P* value < 0.05, — logFC —> 2 were screened, and the R package was applied for gene ontology (GO) enrichment and the Kyoto Encyclopedia of Genes and Genomes (KEGG) pathway analysis. The differential gene protein–protein interaction (PPI) network was constructed using the STRING Cytoscape application, which conducted visual analysis to create the key function networks and identify the key genes. Next, the Kaplan–Meier plotter survival analysis tool was employed to analyze the survival of the key genes derived from the PPI network. Further analysis of the key gene expressions in gastric cancer and normal tissues were performed based on The Cancer Genome Atlas (TCGA) database and RT-qPCR verification.

**Results:**

After transfection of AGS cells, the cell morphology changes in a hummingbird shape and causes the level of CagA phosphorylation to increase. Transcriptomics identified 6882 DEG, of which 4052 were upregulated and 2830 were downregulated, among which q-value < 0.05, FC > 2, and FC under the condition of ≤2. Accordingly, 1062 DEG were screened, of which 594 were upregulated and 468 were downregulated. The DEG participated in a total of 151 biological processes, 56 cell components, and 40 molecular functions. The KEGG pathway analysis revealed that the DEG were involved in 21 pathways. The PPI network analysis revealed three highly interconnected clusters. In addition, 30 DEG with the highest degree were analyzed in the TCGA database. As a result, 12 DEG were found to be highly expressed in gastric cancer, while seven DEG were related to the poor prognosis of gastric cancer. RT-qPCR verification results showed that Helicobacter pylori CagA caused up-regulation of *BPTF, caspase3, CDH1, CTNNB1, and POLR2A* expression.

**Conclusion:**

The current comprehensive analysis provides new insights for exploring the effect of CagA in human gastric cancer, which could help us understand the molecular mechanism underlying the occurrence and development of gastric cancer caused by* Helicobacter pylori*.

## Introduction

Gastric cancer is the fifth-most common malignant tumor and the third-most common cause of death worldwide (Park et al., 2018). The development of gastric cancer involves multiple aspects, including the host factors, environmental factors, and *Helicobacter pylori* infection. Among these, *H. Pylori* infection is known to cause chronic inflammation of the gastric mucosa, which in turn causes atrophic gastritis, gastric cancer, and various other gastrointestinal diseases. Reportedly, *H. Pylori* is a very common infective agent of the stomach across the world, and this infection has been closely related to the development of gastric cancer and its malignant precursors (Valenzuela et al., 2015). Presently, the mechanism of *H. Pylori-* induced damage to the gastric mucosa is not well understood. The assumed possible mechanisms include the damages caused by *H. pylori* colonization and toxin production, the host’s immune response, and the abnormal gastric acid secretion. Accumulating body of work supports that specific virulence factors in *H. Pylori* have a strong correlation with gastric cancer (Chmiela et al., 2017), including CagA and vacuolar cytotoxin A (VacA). CagA is a 128-145-kilodalton (kDa) protein that is composed of a structured N-terminal region and an intrinsically disordered/unstructured C-terminal tail. Variations in the molecular weight of CagA are due to the structural polymorphisms in its C-terminal region, which exist in distinct strains of *H. pylori*. Once injected into the host gastric epithelial cells, CagA is localized to the inner leaflet of the plasma membrane. CagA is encoded by the *H. pylori* cag pathogenic island and injected into gastric epithelial cells via T4SS, where it undergoes tyrosine phosphorylation at the Glu-Pro-Ile-Tyr-Ala (EPIYA) motif in its C-terminal region and then acts as a carcinogenic scaffold protein which physically interacts with different host-signaling proteins. From the sequence flanking the EPIYA motif, four distinct EPIYA segments have been identified in the CagA protein: EPIYA-A, EPIYA-B, EPIYA-C, and EPIYA-D.The EPIYA-C segment is present in variable numbers of copies among distinct Western CagA variants, typically represented in tandem between one to three times. The EPIYA-repeat region of CagA found in East Asian countries also possesses EPIYA-A and EPIYA-B segments but,instead of the tandem EPIYA-C segment, contains a distinct EPIYA-containing segment termed EPIYA-D (47 amino acids), and the CagA protein is referred to as East Asian CagA or ABD-type CagA.Due to the variation of the sequence flanking the tyrosine (Y) residue, the distinct EPIYA segments are tyrosine-phosphorylatedselectively by different kinases.EPIYA-A and EPIYA-C or EPIYA-B and EPIYA-D are preferably phosphorylated in combination in Western CagA and East Asian CagA, respectively.Therefore, there may be a stepwise event in which EPIYA-C or EPIYA-D is phosphorylated by SFKs at the start of an infection followed by phosphorylation of EPIYA-A or EPIYA-B by c-Abl at a subsequent time .Deregulation of SHP2, the pro-oncogenic PTPase involved in the regulation of cell growth, motility, and morphology.East Asian CagA exhibits a stronger ability to bind/deregulate SHP2 and a greater capability to induce SHP2-dependent morphological changes in gastric epithelial cells than Western CagA. Collectively, the findings reveal that the East Asian CagA-specific EPIpYA-D motif is qualitatively very different from the Western CagA-specific EPIpYA-C motif in terms of the biological activity required for deregulation of the SHP2 oncopro-tein, which may causatively account for the higher incidence of gastric cancers in East Asian countries than in Western countries (Takahashi-Kanemitsu, Knight & Hatakeyama, 2020). CagA affects the proliferation and apoptosis of cells through various regulation and signaling pathways, ultimately promoting gastric mucosal carcinogenesis (Takahashi-Kanemitsu, Knight & Hatakeyama, 2020).

Past studies have demonstrated that the non-physiological scaffolding of CagA in cells promote the malignant transformation of normal cells by conferring onto them cancer markers with multiple phenotypes. In chronic inflammation, CagA’s *in vivo* carcinogenic activity is further enhanced. Because *H. pylori* infection triggers a pro-inflammatory response in the host cell, the resultant feed-forward stimulation loop enhances the carcinogenic effects of CagA and cause inflammation in the gastric mucosa, where CagA is injected. Considering the need for clarification on these aspects, we attempted to explore the molecular mechanisms of CagA-induced gastric epithelial cells to seek effective molecular targets in order to provide a basis for early clinical diagnosis, prevention, and treatment of gastric cancer (Cover, 2016). Then, we applied integrated bioinformatics to identify the key genes involved in the process of CagA-induced gastric epithelial cell inflammation and canceration to comprehend the potential molecular mechanisms involved.

## Materials and Methods

### pcDNA3.1::CagA plasmid vector transfection of AGS cells

The CagA plasmid pcDNA3.1(+)/cagA and the empty vector pcDNA3.1(+)/EGFP were purchased from Nobel Biotech (Shanghai, China). AGS cells were obtained from the ATCC. AGS cells were incubated in RPMI-1640 medium (Gibco, Grand Island, NY, USA) supplemented with 10% heat-inactivated fetal bovine serum (Gibco), 100 U/ml of penicillin, and 100 g/ml of streptomycin at 37 °C in a humidified incubator (NSE, Brunswick, NJ, USA) containing 5% CO_2_. AGS cells were seeded in 6-well plates respectively at a density of 5 × 10^6^ cells/well, grown to whose confluence reached at 60–70%, then the cells were transfected with 3 µg plasmid and 5 µl Lipofectamine 2000 (Invitrogen, USA) in 125 µl Opti-MEM™ medium (Gibco, USA) followed by the addition of 1,875 µl Opti-MEM™ medium according to the manufacturer. After 24 h the transfection efficiency was evaluated by observation under a fluorescence microscope, and the relevant cell samples were collected. The CagA expression was verified by western blotting.

### Differential gene collection and screening

The vectors pcDNA3.1::CagA and pcDNA3.1 were transfected into AGS cells respectively. After 24 h, cell samples were collected and sent to NOVOgene (Beijing, China) transcriptome for sequencing to obtain the differentially expressed genes between the two. By adjusting *P* < 0.05, — logFC —>2, the genes with significant differences are listed.

### Analysis of gene ontology (GO) enrichment and the Kyoto Encyclopedia of Genes and Genomes (KEGG) pathway of differentially expressed genes (DEG)

The GO enrichment analysis is a commonly used method for large-scale functional analysis. The gene functions can be classified as biological processes (BP), molecular functions (MF), and cellular components (CC). KEGG is a widely used database that stores the information on a large number of related genomes, biological pathways, diseases, chemicals, and drugs. We applied the R package with data package, visualization, and integrated discovery to perform GO enrichment analysis and KEGG pathway analysis on the DEG in this study, with *P* < 0.05 considered as statistically significant, and passed the “ggplot2” of R package to visually generate histograms and lists (Tang et al., 2020).

### PPI network construction and key cluster identification

The PPI networks of DEG were constructed using the STRING database (Gu et al., 2019) (http://string-db.org), which is a software application that is commonly used to identify interactions, assess potential PPI relationships, and identify previously determined differences. Briefly, the DEG were mapped into the STRING database. The PPI networks were then visualized by the Cytoscape software (Gu et al., 2019) (https://cytoscape.org/). The software predicts the network, with each node as a gene. The network visualization helps identify the interactions and pathway relationships among the proteins encoded by DEG in gastric cancer. The corresponding protein in the central node could be a core protein or a key candidate gene with important physiological regulatory functions. According to the Cytoscape visualization network of molecular interactions, the Molecular Complex Detection (MCODE) plug-in is used to identify densely interconnected clusters, based on the following selection criteria: degree ≥ 2, node score ≥ 0.2, K-core ≥ 2, max depth = 100 (Li, 2019).

### Selection of key genes and their expression analysis in Hp infection status

The top 30 central genes with the most connections in the PPI network are defined as key genes. The differential expression of Hp infection and uninfected tissues was analyzed with reference to the TCGA database (*P* < 0.05 is considered to indicate statistical significance).

### Survival analysis of the key genes

The Kaplan–Meier plotter database (Ma, Zhou & Zheng, 2020) (http://www.kmplot.com) is an online tool that can be used to evaluate 54,675 genes under the conditions of 10,461 cancer samples. We used this database to perform a survival analysis (*P* < 0.05 was considered to indicate statistical significance). Functional enrichment analysis of the genes that were highly expressed in gastric cancer was performed.

### Identification of RT-qPCR

The cagA gene knockout mutant strain Hp/cagA cm was constructed by Sangon Biotech( Shanghai, China). AGS cells were seeded in 6-well plates respectively at a density of 5 × 10 ^6^ cells/well, grown to whose confluence reached at 60–70%, then the cells were infected with Hp/ ΔcagA::Cm and Wild type Hp/cagA ^+^ with a multiplicity of infection (MOI) of 30, respectively. 24 h later, these cells were harvested to investigate BPTF, CASP3, CDH1, CTNNB1 and POLR2A mRNA levels by RT-qPCR.

### Western blot

The total protein was extracted according to the instructions of the lysate kit. After quantification by the BCA protein quantification kit, SDS-PAGE electrophoresis for 2 h, membrane transfer for 2 h, 1XTBST (0.05% Tween20) solution containing skimmed milk powder was blocked at room temperature for 2 h, and CagA primary antibody was added (1: 1,000), p-CagA (1: 1,000), GAPDH (1: 5,000) Incubate overnight at 4 °C, wash the membrane with 1 ×TBST (0.05% Tween20) solution 3 times, 10 min/time, add two Incubate at room temperature for 2 h with anti (1 :10,000), wash the membrane with 1 ×TBST as above, add a chemiluminescence reagent for color development, and expose and image with a chemiluminescence imager.

## Results

### Transfection of pcDNA3.1::CagA plasmid into AGS cells and verification by western blotting

**Figure 1:**
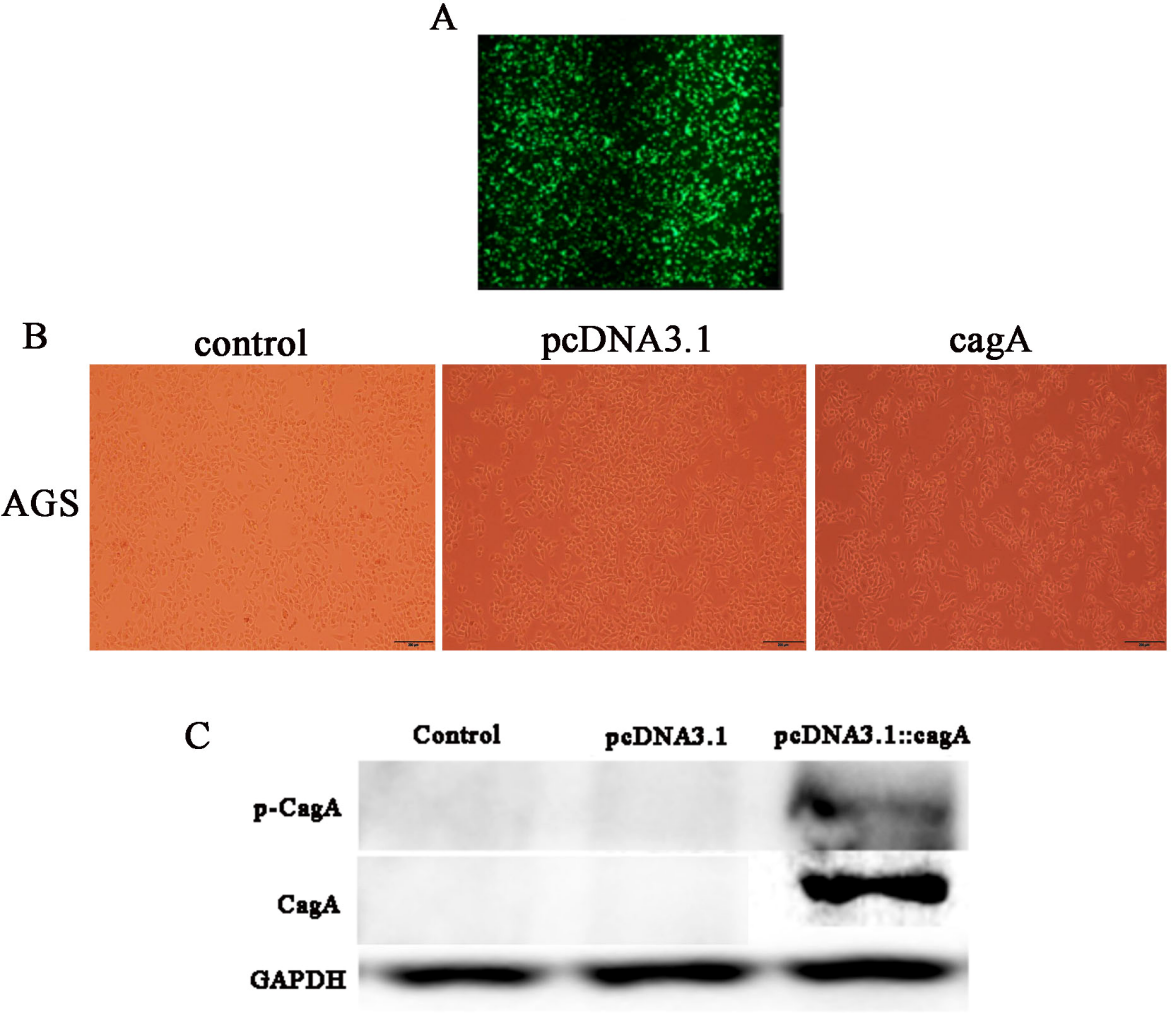
Detection of pcDNA3.1::cagA plasmid transfection efficiency and CagA expression in AGS cells. pcDNA3.1:: cagA plasmid was transfected into AGS cells for 24 h, the transfection efficiency observed under a microscope and measure the protein levels of CagA and phosphorylated CagA by Western blotting. (A) Fluorescence showed that the transfection efficiency reached > 70%; (B) cagA plasmid transfected group shows hummingbird-like changes in cell morphology. (C) The protein levels of CagA and p-CagA were showed inpcDNA3.1::cagA transfected group. The control group represents the untreated group; pcDNA3.1 group represents the AGS cells transfeced with empty vecter pcDNA3.1; pcDNA3.1::cagA group represents the AGS cells transfected with pcDNA3.1:: cagA.

After 24 h of transfection of pcDNA3.1::CagA, The efficiency of fluorescent transfection is estimated to be >70% (Fig. 1A). The morphology of the cells was observed under the microscope. It was found that compared with the control group(Control) and the empty vector group(pcDNA3.1), the morphology of the cells in the cagA transfection group(cagA) changed significantly. The shape of the cell changed from obtuse to long fusiform, spindle-shaped, irregular, and the polarity of the cell disappeared, showing a ‘hummingbirdchange’ (Fig. 1B). Western blot verification showed that CagA and p-CagA protein was successfully expressed in pcDNA3.1::cagA transfected group (Fig. 1C).

**Figure 2:**
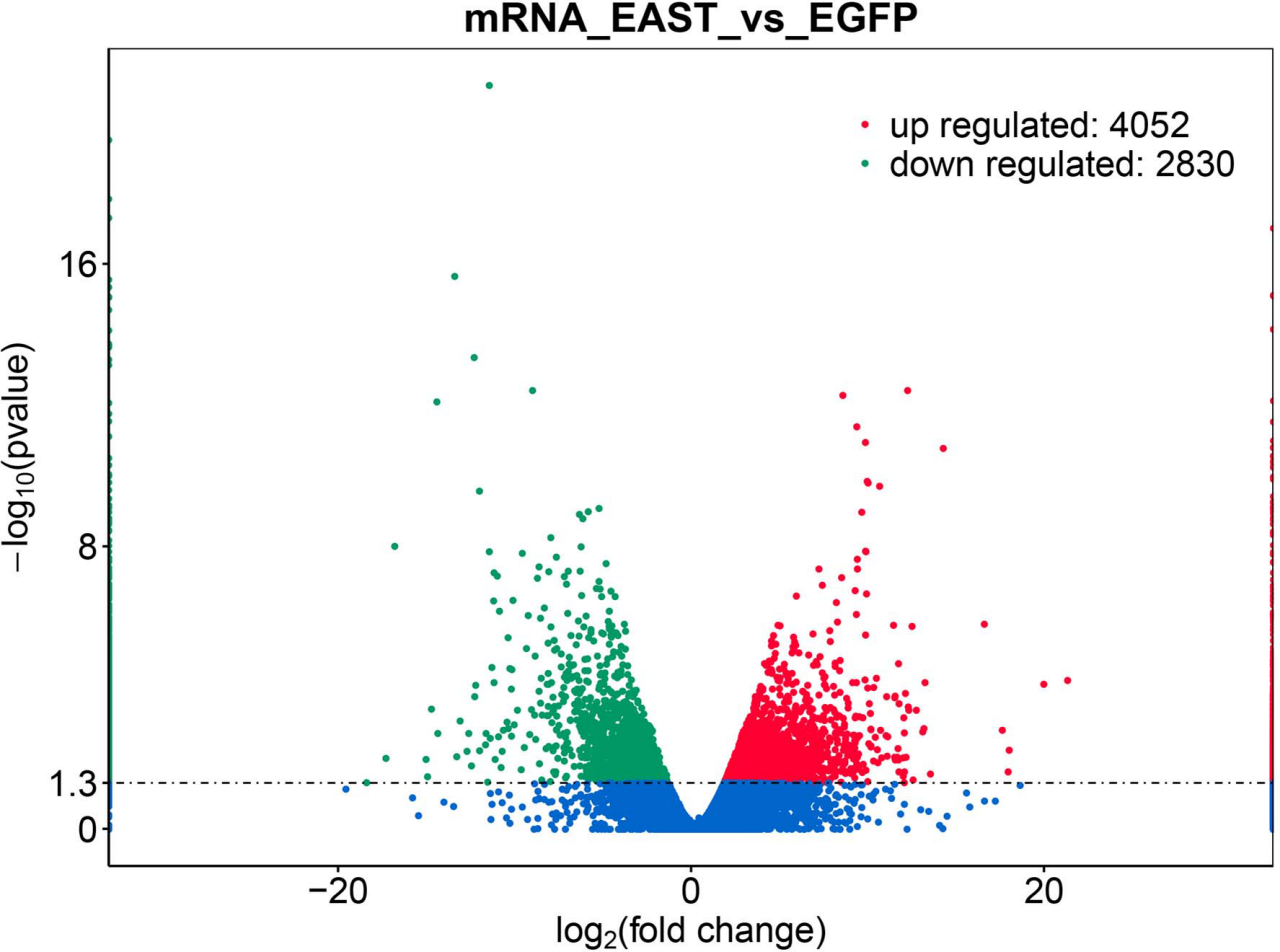
Differential expression of data between two sample sets. The red points represent upregulated genes screened on the basis of |fold change |>2.0 and a corrected *P* value of <0.05. The green points represent downregulation of the gene expression screened on the basis of |fold change |>2.0 and a corrected *P*-value of <0.05. The blue points represent genes with no significant difference. FC indicates the fold change.

**Table 1:** Filter out statistically significant DEGs.

**DEGs**	**Gene names**
**Upregulated:**	ATM AHNAK2 MYCBP2 UBE4A KIAA1109 MIEF1 LSM12 FRYL SYVN1 GSN RERE STK36 ACTN1 SMG1 KMT2A PIK3CB HAS3 ACOT7 CHTF8 KIF21B ANO1 ADCY3 PPIP5K1 HUWE1 ZNF184 ADGRG1 ABR MUC1 FRYL PTPRF KMT2D BICRA DHCR24 TNRC18 BCORL1 YLPM1 SYNE1 DST YLPM1 COL1A1 XIAP NCOR2 BRD4 TRIM32 CTDP1 HSPA8 EIF3L PRR12 KDM6B LMNA NUP214 LAMA5 CKMT1A ARHGEF11 ATXN2L HSPG2 NCOA6 BAZ2A PIGB BPTF AKT1S1 PCBP2 MCM3AP NR2F2 ATN1 MYO10 AFF1 FNDC3A ILF3 VPS13D FGD4 MYSM1 NOL8 LAS1L ANP32E FOXM1 DGKZ CDC73 CALU SUPT20H GLT8D1 SLCO2A1 NEDD4L OXA1L CTNNA1 PCBP2 GART ALG11 RREB1 DDX5 PTCH1 TYW3 ZNF703 TJP2 HMBOX1 C6orf106 NPIPB12 CLASP1 ERGIC3 PEAK1 LRP1 ARNTL CTNNB1 DDX11 SCMH1 ZNF615 DENND3 RAPH1 KMT2D TRIM16 SMC4 SUPT5H SLC39A4 YWHAZ ZFP36L1 LRRC8D MED13L POLR2A SRCAP SMC1A TRMT1 PEX5 PARVA BIRC2 LCOR TNK2 BCL9L CTNND1 RARG AAK1 TENT2 CACHD1 ZC3HAV1 UBE2V1 UBR4 CEP85 SPEN PDXDC1 DNM2 AKAP13 DLGAP4 PTPN23 MLXIP STAT1 CAP1 CAMSAP2 ADNP SRCAP KANSL1L TMEM150A ARHGEF10L TBC1D15 SH3D19 PRRC2C SRRM2 MLXIP NPIPB5 P2RX4 TEF PPP6C ZNF615 ENTPD2 PROX1 VKORC1L1 EP400 SH3YL1 DPF2 SEPT2 MUC1 RABEP2 ZNFX1 SLC9A7 PGRMC2 CPEB3 TCTN3 RAD23B KDM6B CELSR2 WIZ NRDC CEACAM1 YAP1 RALBP1 SEC31A UBAP2L STAT6 CLTC FOXP1 FBXW11 CNOT6 KMT2C ARHGEF11 SMARCA4 TBC1D7 ZSWIM8 ABTB1 POM121 VPS37B CLASP2 SRGAP1 SLC35A3 RNPS1 SLC30A6 SEPT9 CCNE2 USP20 TGFBRAP1 RPL21 STX16 BCAS3 RBM26 CREBBP HDAC1 BTBD3 URGCP FOXP1 CSDE1 ZNF28 PKIG RPS7 IFT140 SRCAP GSN SECISBP2 LTBP3 TJP2 GPR35 PRSS23 PLA2G2A DENND4B CREBBP ASXL2 CABIN1 ESYT2 ATP2A2 MYCBP2 PRAG1 KANSL1 SORBS2 ARID1A MEF2D RPL34 RALGPS1 CEP85 CBFA2T2 TACC2 ZNF10 IGF1R HDAC8 ACIN1 ZFAND6 ZFHX3 HNRNPH3 ZMIZ1 RHOC ZNF638 SYNPO TPK1 HNRNPA3 SSBP3 ATG2A PPP4C PLEC IGSF9 IP6K1 NFATC3 PIGN SAMD12 VOPP1 AC091057.6 ASPSCR1 HNRNPA3 HECTD4 ATXN2L DOCK9 PLXNB2 CEP44 NPIPB3 FGFR2 AP3B1 MYO10 ZNF720 HARS EPB41L2 SEPT7 RNF145 MPND PLCXD1 MEST MX2 ZNF567 CACNG8 KMT2D KAT6B PPM1B FASN RERE RHBDF2 RNF128 SFT2D2 DISC1 TANC2 APBB2 MED12 ARID1A PIK3CB ZFPM1 GRIN2D ZFHX2 FGGY LEPR NPIPB4 C6orf132 TRIOBP ANKRD11 OXSR1 FAM210A SMIM8 FAM98B STK24 AFDN AHDC1 AL354822.1 AFAP1 FUS TBC1D15 HNRNPA2B1 SEPT9 NKD1 YIPF1 TSPAN14 ADAR ESRP1 NPIPB5 ATXN10 BRD3OS KMT2B DYNC2H1 TRIM7 USP48 DMTN BCL11B COBL ZC3H12C FAM47E XBP1 PCBP2 R3HCC1L MRTFA ARHGEF9 ERI1 RNF4 CASP3 EML4 PTPN21 RNF213 ATRX SYNE2 SCAF1 BROX DSP NPIPB5 SMARCAD1 USP48 ASCC1 ZNF107 DCAF13 PDCD4 PIK3C2B HCFC1 USP49 ANO1 ARHGAP12 MEN1 TAF1C PDX1 SAMD4B MYBBP1A NEK11 TAB2 MAVS NEURL4 KDM6A ELMSAN1 PILRB ZNF532 TRIM66 VPS35L TXNDC11 ZFHX3 PHLDB2 MYO10 NLRP1 MATN2 ANP32B TRIM27 RNF111 HNRNPH2 EP400 PARP9 CNOT9 HOXC6 ZNF761 LARP7 NR4A1 GAA ZNF841 SMC1A RNPS1 MBNL2 PHTF1 YLPM1 PTPN12 IPO8 VTA1 CHD4 EPS8 PTK2 FBXW7 MICAL3 MAST4 PITRM1 DCLRE1C PTOV1 ARMCX4 TIMP1 HELZ2 KIAA0895 CDH1 LTN1 ZC3H4 ZNF865 HNRNPM TNKS AAK1 CMIP ZNF326 CTBP2 CFAP44 SH3D19 PKP4 TRRAP HELZ2 TRIM4 LITAF SLC22A18 MUC1 UBE2D2 PPARG RUVBL1 SLC22A23 ATRX TMEM214 UCKL1 TTC21A KIAA1211 NPIPB3 PCM1 SMARCA4 PLXNB2 KDM4B HNRNPUL1 POM121C MEST BCL11A SMAD5 ACVR1B RANGAP1 SEC16A CEACAM1 RAB7B ADCY3 GRK6 MORC2 RBM17 NAV2 ANKFY1 SON FBXL8 CDRT4 RRM2 PLCB1 PCDHGB5 CRTAP CREBBP URM1 CANT1 DCAKD HIPK2 PLEC ZNF18 SMARCA4 AHNAK MEF2D ALG11 C1QTNF3 HNRNPU SEZ6L2 BCAM LIMA1 USP6NL ELF1 AFDN ARID4B PABPC4 PTPRF CCNK WDHD1 UBE2K SH3D19 BCLAF3 USP16 PRDM2 HADH ADAM20 GEMIN2 GRAMD1A POU5F2 ZNF516 PLEKHA7 TMEM80 HTATSF1 PHKB MIDN CREB3L2 SP110 KCNC3 PCDHGA12 TSFM BCL9 COL27A1 BRD1 KDM6A TCTN1 KMT2A SETD1A RAB12 AP3D1 MICAL1 MROH1 RBM33 CPT2 MEIS2 ITGB1 TGFB1 COPZ1 KRBA2 ST3GAL3 MPLKIP TMCC3 ZBTB43 NCOR2 RAVER1 ZBED3 EPHX1 FAS KDM4C USP48 KIAA1217 NFAT5 PCMTD2 NPIPB3 SOCS4 PPP6C UVSSA HMGA2 MYCBP2 TAP2 RUNX1 TRIO ABCB8 LTBP3 MMS19 TAF5L USP21 KIAA1549 DIDO1 GRAMD1A PLEKHB2 FTO CLK2 MAML1 ITSN1 RNF4 TRIM3 SUCLA2 RPL18 HNRNPD PML CHD1 DTX2 RUNX1 PALLD TVP23C
**Downregulated:**	DHCR24 GRSF1 MRNIP RALBP1 HACD3 RNF6 LSM7 MATR3 PDIA6 TAF5L RPL15 HBP1 IFNGR1 NEDD1 SCAND1 TCEA1 SLA2 PSMC2 CNTD1 SNX22 FAM161B ISG15 CAPN12 NACA BEST1 ACSBG1 RPS27 FAM166A CEP85 SAP18 MORF4L1 KMO ARFGAP1 MMACHC NOL8 EML5 TYK2 ATP5ME CPM GSTM3 EXOC3 MXD3 SLC9A6 PLEKHB2 PHTF1 CCDC73 TUBB6 TMEM87A LTO1 RPL34 TAGLN SRRT PET100 ATP6V1C2 MCF2L2 RPL5 AC025283.2 AC243967.1 LSMEM2 STX6 SSR1 MPC2 SLC4A11 SCAND1 CIDEB FARP1 LCOR TGFB1 RAB11A GLIPR1 RSPH10B RPL27 SARM1 IMPA1 ATXN7L3 USP33 PFDN5 CASP4 MPLKIP HNRNPDL ZNF283 NR1I3 USP33 STRADB ATM GAN ASAH1 ATP5MF SERF2 ATP2B1 TBCB RBM6 LRPAP1 IFNLR1 DTNBP1 PFKFB2 LGR5 FAM220A WDR83OS AC015802.6 NECAP1 CCSAP BCAP31 RPL36 C19orf53 BCAT2 AHNAK2 SLC18A2 UGDH HIST1H2BC UBAP1 HNRNPAB CDK4 SKP1 GLOD4 UBXN1 NDUFS5 NDUFB2 TMEM222 SLC9A6 ZNF75A TRIM59 RPL35A CCDC12 GPS1 CSNK2A1 PCF11 RILP FAR2 METTL6 PLLP RBPJ ZFAND6 TSPAN31 CPSF6 ETFRF1 RBM42 CASKIN1 DENND1C PRKAR1B CLDN6 DNAH11 FAM222B LBHD1 LARS DYNC1I2 RAN KPNB1 TJP2 MYL6 CDC42 KLC4 ANKS1B UGP2 ATP5MC2 NKX6-2 ATP8B4 TOMM5 SGK3 VPS35L ZNF143 PFDN6 NDUFA1 TBC1D31 ELOC RALB COX7A2 HM13 GSTP1 IGFBP7 PMFBP1 MAP2K4 SMCO4 SPC24 NR1I3 SAP18 SHLD2 FAM92A AL391650.1 RPL23 FUZ BNIP2 C11orf98 PARD3 CBFB AC138811.2 FLG DENND2A PARD3 NKAPD1 EMILIN3 MMADHC SPTY2D1OS IDI2 SLBP COQ8B CABP7 SEMA4D RPL38 FSD2 B3GAT2 NAP1L4 NDUFB1 KIAA0895 SNAPC5 IL17D UBA3 IFT20 NDUFS6 HARS2 EVPL RAD51AP1 MPC2 DOK7 TMBIM6 FASTK TATDN1 TNKS1BP1 ATP5MF RPL27 ZACN OR2I1P SERPINA1 MRPL13 PMEL UBP1 SMIM12 COCH CP SLC30A5 RNF4 GUK1 DCDC2B TRIM37 SLC39A6 UBP1 ASNSD1 TRMT112 POC1B-GALNT4 TMCO1 SLC35B1 PSMC3 GCA TMEM200B EDF1 C19orf53 ARPC5L NOTCH2NLA TMA7 PHTF2 FAM177A1 MTLN NAP1L1 AHCTF1 NDUFS5 HMGN2 RPL37A CBWD6 GTF2A2 TAF1D GPM6B FBXL17 HARS PRMT7 GMFG RPL35A CENPW PEX5 INTS9 PPP4C RPL11 ATG4C TFEB RPL21 PRR13 CBLN3 GPR22 NACA RPL36A TAP2 HSFX4 CFAP36 COX14 HIST1H3J UQCRB CEP57 RPL37 YAP1 ODC1 FANCL TMEM223 RPL31 HMOX2 SRP14 HOMER3 CFAP73 TUBB1 RUVBL1 TPM1 TOMM7 AC001226.2 KAT6A IFNGR1 PPP1R18 AC092718.8 CCDC175 DHCR24 ZNF320 PRAG1 RAD52 PARD3 PFDN5 DOC2A PDCD5 HARS SEPT2 HIST1H4L MRNIP PFN1 BTF3L4 CIRBP RPL30 SYNC GPI TCEAL4 PTGDR2 NPC2 ERLIN2 RSPH9 RNF38 ENTPD1 MEMO1 ESRP1 ANKRD6 UBA52 RPL21 SMARCE1 AFDN SLC35A2 CD72 CD63 PHB KRT8 PI4KA GMFB NAA38 RPL38 DRAP1 ESRP1 OCIAD1 RAB12 PPP1R27 ENY2 SYK DNAAF1 PKM PRELID1 TRMT112 SLC19A2 UBA52 PTPN2 NRF1 PAGE4 TMEM33 PPP1R13B AMZ2 MRPL33 RPS23 TCP11L1 XRCC3 ZNF74 UBXN1 PRDX5 C8orf59 CFAP57 CAST WNT5B CSNK2B SPAG9 DAZAP2 SH3YL1 RPS2 ARMCX3 PON2 RPL36A DMTN RPL13 SCARB2 S100A2 HSFX3 RAP1GAP KDELR1 C7orf25 COA6 ACADM HARBI1 C5orf30 EFCAB2 PTGES3 PHKG1 PDCD10 GPAT4 HACD4 PAFAH1B1 MYL6 CD55 LONP1 RPL13A BCAP31 SLC5A2 C2orf16 UQCRC2 IFNGR2 SRPX2 SLC10A1 CBFA2T2 EFNB2 STUB1 HADHA DHCR24 EFCAB10 IMMT AMN1 NDRG1 RPL8 ZFAND6 PTMA TULP2 SERPINB6 M6PR RPL28 AGR2 HDAC4 SPNS2 MID1IP1 CAST EXOSC8 CTDNEP1 CGGBP1 SLC29A1 USP45 ASCC2 SLC7A7 ATP6V1G2 C7orf25 FAM81A SH3GLB1 OSBPL2 CPOX ALKBH6 SOD2 STRBP NAT9 GCSAM LSS ZNF3 TMEM218 SERINC4 ORMDL1 1-Mar MECP2 DBI MMEL1 LRP6 TAF1C DALRD3 RPS6KA3 RPL35

**Notes.**

Abbreviations DEGs differentially expressed genes

**Figure 3:**
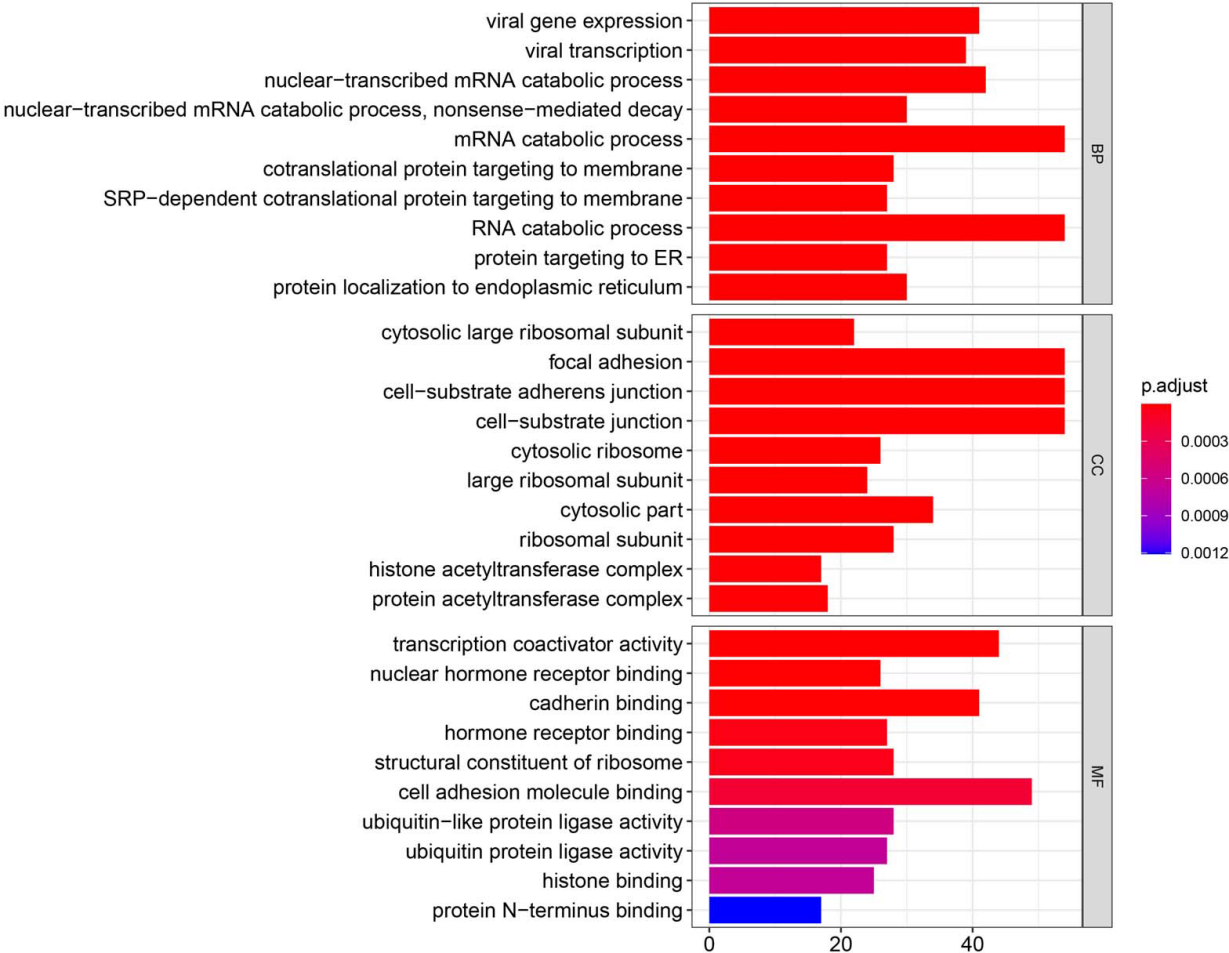
GO enrichment analysis of DEGs. GO analysis categorized DEG into three functional groups: molecular function, biological processes, and cell composition.

### Screening DEG

The application of transcriptomics identified 6,882 common genes, of which 4052 were upregulated and 2830 were downregulated (Fig. 2). At adjusted *P* < 0.05, —logFC —>2, 1062, DEG with statistical significance were further screened to identify 594 upregulated and 468 downregulated genes (Table 1).

**Table 2:** GO analysis of DEGs.

**Term**	**Description**	**Count**	*P*-value
GO:0019080	viral gene expression	41	2.00E–13
GO:0019083	viral transcription	39	2.30E–13
GO:0000956	nuclear-transcribed mRNA catabolic process	42	2.37E–13
GO:0000184	nuclear-transcribed mRNA catabolic process, nonsense-mediated decay	30	1.00E–11
GO:0006402	mRNA catabolic process	54	1.00E–11
GO:0006613	cotranslational protein targeting to membrane	28	2.82E–11
GO:0006614	SRP-dependent cotranslational protein targeting to membrane	27	6.77E–11
GO:0006401	RNA catabolic process	54	2.36E–10
GO:0045047	protein targeting to ER	27	1.09E–09
GO:0070972	protein localization to endoplasmic reticulum	30	1.37E–09
GO:0072599	establishment of protein localization to endoplasmic reticulum	27	2.08E–09
GO:0016569	covalent chromatin modification	57	5.86E–09
GO:0016570	histone modification	55	9.37E–09
GO:0006612	protein targeting to membrane	33	4.53E–08
GO:0006605	protein targeting	51	1.11E–07
GO:0006338	chromatin remodeling	28	3.94E–06
GO:0090150	establishment of protein localization to membrane	40	4.60E–06
GO:0019058	viral life cycle	39	9.44E–06
GO:1904837	beta-catenin-TCF complex assembly	11	9.72E–06
GO:0006413	translational initiation	28	1.16E–05
GO:0030522	intracellular receptor signaling pathway	33	0.000112261
GO:0043401	steroid hormone mediated signaling pathway	25	0.000193979
GO:0071383	cellular response to steroid hormone stimulus	30	0.000231361
GO:0018205	peptidyl-lysine modification	40	0.000377348
GO:0031647	regulation of protein stability	32	0.000377348
GO:0030099	myeloid cell differentiation	40	0.001113214
GO:0034332	adherens junction organization	20	0.001117253
GO:0050792	regulation of viral process	25	0.001423449
GO:0009755	hormone-mediated signaling pathway	27	0.001423449
GO:0043484	regulation of RNA splicing	19	0.001726434
GO:0043900	regulation of multi-organism process	38	0.002773222
GO:0034330	cell junction organization	30	0.003325059
GO:0043903	regulation of symbiosis, encompassing mutualism through parasitism	25	0.003664061
GO:0016573	histone acetylation	20	0.003664061
GO:0030518	intracellular steroid hormone receptor signaling pathway	18	0.003887898
GO:0019079	viral genome replication	17	0.004734418
GO:0048525	negative regulation of viral process	15	0.004893681
GO:0018393	internal peptidyl-lysine acetylation	20	0.005107113
GO:0043967	histone H4 acetylation	12	0.005107113
GO:0002181	cytoplasmic translation	15	0.005107113
GO:0033143	regulation of intracellular steroid hormone receptor signaling pathway	13	0.005220493
GO:0016331	morphogenesis of embryonic epithelium	19	0.00555893
GO:0016578	histone deubiquitination	7	0.005647965
GO:0051052	regulation of DNA metabolic process	38	0.006801205
GO:0006475	internal protein amino acid acetylation	20	0.006801205
GO:1905331	negative regulation of morphogenesis of an epithelium	6	0.007269559
GO:0042692	muscle cell differentiation	35	0.007269559
GO:0048545	response to steroid hormone	35	0.007269559
GO:0030521	androgen receptor signaling pathway	11	0.007885335
GO:0018394	peptidyl-lysine acetylation	20	0.007885335
GO:0072175	epithelial tube formation	17	0.009185473
GO:0034329	cell junction assembly	25	0.009304069
GO:1903901	negative regulation of viral life cycle	13	0.010119749
GO:0030111	regulation of Wnt signaling pathway	33	0.010318481
GO:0035148	tube formation	18	0.011328993
GO:0006473	protein acetylation	22	0.012697644
GO:0044782	cilium organization	34	0.01287833
GO:0006354	DNA-templated transcription, elongation	15	0.013214194
GO:0030177	positive regulation of Wnt signaling pathway	20	0.014794079
GO:0006984	ER-nucleus signaling pathway	9	0.014859166
GO:0045637	regulation of myeloid cell differentiation	25	0.014859166
GO:0008380	RNA splicing	39	0.014859166
GO:0034968	histone lysine methylation	15	0.014859166
GO:0001843	neural tube closure	13	0.014920111
GO:0034333	adherens junction assembly	13	0.014920111
GO:0051147	regulation of muscle cell differentiation	20	0.01537082
GO:0060606	tube closure	13	0.016202371
GO:0051348	negative regulation of transferase activity	27	0.01736378
GO:0060765	regulation of androgen receptor signaling pathway	7	0.017685114
GO:0001841	neural tube formation	14	0.018715149
GO:0032204	regulation of telomere maintenance	12	0.018715149
GO:0043901	negative regulation of muxlti-organism process	19	0.018715149
GO:0051054	positive regulation of DNA metabolic process	23	0.018715149
GO:0060271	cilium assembly	32	0.019274508
GO:0060070	canonical Wnt signaling pathway	30	0.019418493
GO:0021915	neural tube development	18	0.02189728
GO:0010171	body morphogenesis	9	0.022689191
GO:0032784	regulation of DNA-templated transcription, elongation	9	0.022689191
GO:1903311	regulation of mRNA metabolic process	29	0.02307114
GO:0044319	wound healing, spreading of cells	7	0.02307114
GO:0090505	epiboly involved in wound healing	7	0.02307114
GO:2000781	positive regulation of double-strand break repair	7	0.02307114
GO:0060766	negative regulation of androgen receptor signaling pathway	5	0.02307114
GO:0000723	telomere maintenance	18	0.02307114
GO:0000209	protein polyubiquitination	28	0.023399292
GO:1903900	regulation of viral life cycle	17	0.023399292
GO:0007044	cell-substrate junction assembly	13	0.023399292
GO:0014020	primary neural tube formation	13	0.023399292
GO:0090504	epiboly	7	0.026209669
GO:0001838	embryonic epithelial tube formation	15	0.026209669
GO:0006479	protein methylation	19	0.026209669
GO:0008213	protein alkylation	19	0.026209669
GO:0036124	histone H3-K9 trimethylation	5	0.028504092
GO:0016571	histone methylation	16	0.028504092
GO:0034976	response to endoplasmic reticulum stress	26	0.028504092
GO:0036498	IRE1-mediated unfolded protein response	10	0.028504092
GO:0046782	regulation of viral transcription	10	0.028504092
GO:0001837	epithelial to mesenchymal transition	16	0.02997652
GO:0072665	protein localization to vacuole	10	0.031694802
GO:0071824	protein-DNA complex subunit organization	26	0.031924183
GO:2000779	regulation of double-strand break repair	11	0.032301863
GO:0016049	cell growth	38	0.033335915
GO:0018023	peptidyl-lysine trimethylation	8	0.033675333
GO:0060330	regulation of response to interferon-gamma	6	0.033675333
GO:0060334	regulation of interferon-gamma-mediated signaling pathway	6	0.033675333
GO:0018022	peptidyl-lysine methylation	15	0.0339268
GO:0002011	morphogenesis of an epithelial sheet	9	0.034329329
GO:1903391	regulation of adherens junction organization	10	0.037116501
GO:0051098	regulation of binding	31	0.037419782
GO:0034504	protein localization to nucleus	24	0.037419782
GO:0033144	negative regulation of intracellular steroid hormone receptor signaling pathway	7	0.037419782
GO:0043543	protein acylation	23	0.039774544
GO:0055007	cardiac muscle cell differentiation	15	0.040008254
GO:1903706	regulation of hemopoiesis	37	0.040008254
GO:0006913	nucleocytoplasmic transport	29	0.040008254
GO:0006352	DNA-templated transcription, initiation	23	0.040379329
GO:0002067	glandular epithelial cell differentiation	8	0.040379329
GO:0007041	lysosomal transport	13	0.040379329
GO:0032200	telomere organization	18	0.040379329
GO:0051895	negative regulation of focal adhesion assembly	5	0.040379329
GO:0097242	amyloid-beta clearance	7	0.040379329
GO:0031503	protein-containing complex localization	25	0.040379329
GO:0007045	cell-substrate adherens junction assembly	11	0.040379329
GO:0048041	focal adhesion assembly	11	0.040379329
GO:0060560	developmental growth involved in morphogenesis	22	0.040379329
GO:0051169	nuclear transport	29	0.040822401
GO:0010172	embryonic body morphogenesis	4	0.040822401
GO:0048096	chromatin-mediated maintenance of transcription	4	0.040822401
GO:0070933	histone H4 deacetylation	4	0.040822401
GO:1900112	regulation of histone H3-K9 trimethylation	4	0.040822401
GO:0043921	modulation by host of viral transcription	6	0.04145549
GO:0052472	modulation by host of symbiont transcription	6	0.04145549
GO:0001959	regulation of cytokine-mediated signaling pathway	18	0.041669901
GO:0071156	regulation of cell cycle arrest	13	0.042842707
GO:2000058	regulation of ubiquitin-dependent protein catabolic process	16	0.042842707
GO:0042176	regulation of protein catabolic process	31	0.042914363
GO:0006623	protein targeting to vacuole	7	0.042921458
GO:0051261	protein depolymerization 13 0.044702762
GO:0051099	positive regulation of binding	18	0.044702762
GO:0051972	regulation of telomerase activity	8	0.044702762
GO:0072666	establishment of protein localization to vacuole	8	0.044702762
GO:2000059	negative regulation of ubiquitin-dependent protein catabolic process	8	0.044702762
GO:1902115	regulation of organelle assembly	19	0.044826787
GO:0050684	regulation of mRNA processing	15	0.046220077
GO:0052312	modulation of transcription in other organism involved in symbiotic interaction	6	0.046220077
GO:0051893	regulation of focal adhesion assembly	9	0.047313553
GO:0090109	regulation of cell-substrate junction assembly	9	0.047313553
GO:0015931	nucleobase-containing compound transport	22	0.047313553
GO:0051236	establishment of RNA localization	19	0.048534128
GO:0008347	glial cell migration	8	0.048610574
GO:0097193	intrinsic apoptotic signaling pathway	25	0.048695169
GO:0022625	cytosolic large ribosomal subunit	22	7.58E–12
GO:0005925	focal adhesion	54	1.22E–10
GO:0005924	cell-substrate adherens junction	54	1.22E–10
GO:0030055	cell-substrate junction	54	1.35E–10
GO:0022626	cytosolic ribosome	26	2.99E–10
GO:0015934	large ribosomal subunit	24	5.69E–08
GO:0044445	cytosolic part	34	3.89E–07
GO:0044391	ribosomal subunit	28	1.77E–06
GO:0000123	histone acetyltransferase complex	17	9.25E–06
GO:0031248	protein acetyltransferase complex	18	9.25E–06
GO:1902493	acetyltransferase complex	18	9.25E–06
GO:0035097	histone methyltransferase complex	16	5.31E–05
GO:0016363	nuclear matrix	18	6.56E–05
GO:0034399	nuclear periphery	19	0.0002262
GO:0070603	SWI/SNF superfamily-type complex	14	0.0002262
GO:0042788	polysomal ribosome	9	0.000278795
GO:0098984	neuron to neuron synapse	34	0.000596952
GO:0014069	postsynaptic density	32	0.000714655
GO:0032279	asymmetric synapse	32	0.000864164
GO:0030496	midbody	21	0.000875088
GO:0005840	ribosome	28	0.000907132
GO:0000790	nuclear chromatin	35	0.000907132
GO:0099572	postsynaptic specialization	33	0.000926357
GO:1904949	ATPase complex	15	0.000926357
GO:0034708	methyltransferase complex	16	0.000926357
GO:0005667	transcription factor complex	34	0.000926357
GO:0016607	nuclear speck	36	0.000940948
GO:0005938	cell cortex	30	0.001070189
GO:0101002	ficolin-1-rich granule	21	0.001630215
GO:0044798	nuclear transcription factor complex	22	0.001853344
GO:0000812	Swr1 complex	5	0.002106877
GO:0000118	histone deacetylase complex	10	0.004130179
GO:0042470	melanosome	14	0.004493787
GO:0048770	pigment granule	14	0.004493787
GO:0005844	polysome	11	0.005579034
GO:0090575	RNA polymerase II transcription factor complex	18	0.005891446
GO:1904813	ficolin-1-rich granule lumen	15	0.006649382
GO:1902562	H4 histone acetyltransferase complex	8	0.00862081
GO:0031252	cell leading edge	33	0.00862081
GO:0005643	nuclear pore	12	0.00862081
GO:0005635	nuclear envelope	36	0.012865041
GO:0099092	postsynaptic density, intracellular component	5	0.013474404
GO:0030027	lamellipodium	19	0.014296793
GO:0070461	SAGA-type complex	6	0.017456175
GO:0044455	mitochondrial membrane part	21	0.018871465
GO:0099091	postsynaptic specialization, intracellular component	5	0.026165033
GO:0044666	MLL3/4 complex	4	0.026793714
GO:0005913	cell-cell adherens junction	13	0.026869727
GO:0000792	heterochromatin	10	0.028802567
GO:0099738	cell cortex region	7	0.028802567
GO:0031965	nuclear membrane	24	0.041189729
GO:0000124	SAGA complex	4	0.042262875
GO:0071565	nBAF complex	4	0.042262875
GO:0044309	neuron spine	16	0.045301703
GO:0017053	transcriptional repressor complex	10	0.045301703
GO:0097346	INO80-type complex	5	0.04846594
GO:0016605	PML body	11	0.048596868
GO:0003713	transcription coactivator activity	44	1.54E–07
GO:0035257	nuclear hormone receptor binding	26	5.03E–06
GO:0045296	cadherin binding	41	5.22E–06
GO:0051427	hormone receptor binding	27	4.05E–05
GO:0003735	structural constituent of ribosome	28	5.75E–05
GO:0050839	cell adhesion molecule binding	49	0.000164258
GO:0061659	ubiquitin-like protein ligase activity	28	0.000564585
GO:0061630	ubiquitin protein ligase activity	27	0.000675924
GO:0042393	histone binding	25	0.000675924
GO:0047485	protein N-terminus binding	17	0.001209051
GO:0019787	ubiquitin-like protein transferase activity	39	0.001859565
GO:0035258	steroid hormone receptor binding	15	0.001859565
GO:0004842	ubiquitin-protein transferase activity	36	0.004671669
GO:0003730	mRNA 3’-UTR binding	14	0.005656635
GO:0030374	nuclear receptor transcription coactivator activity	11	0.016358235
GO:0031267	small GTPase binding	38	0.016779445
GO:0017016	Ras GTPase binding	37	0.016779445
GO:0044389	ubiquitin-like protein ligase binding	29	0.016779445
GO:0001085	RNA polymerase II transcription factor binding	18	0.017774698
GO:0003714	transcription corepressor activity	24	0.017774698
GO:0033613	activating transcription factor binding	12	0.024777396
GO:0031625	ubiquitin protein ligase binding	27	0.024777396
GO:0042800	histone methyltransferase activity (H3-K4 specific)	5	0.024777396
GO:0003779	actin binding	36	0.026046781
GO:0005088	Ras guanyl-nucleotide exchange factor activity	16	0.026427777
GO:0004402	histone acetyltransferase activity	10	0.026427777
GO:0046965	retinoid X receptor binding	5	0.027715133
GO:0055106	ubiquitin-protein transferase regulator activity	5	0.027715133
GO:0050681	androgen receptor binding	8	0.028197009
GO:0061733	peptide-lysine-N-acetyltransferase activity	10	0.029296991
GO:0042974	retinoic acid receptor binding	6	0.031275892
GO:0005089	Rho guanyl-nucleotide exchange factor activity	11	0.032203632
GO:0016887	ATPase activity	32	0.037839109
GO:0070577	lysine-acetylated histone binding	5	0.038501268
GO:0140033	acetylation-dependent protein binding	5	0.038501268
GO:0070491	repressing transcription factor binding	10	0.043198409
GO:0016922	nuclear receptor binding	5	0.044382275
GO:0001098	basal transcription machinery binding	10	0.044382275
GO:0001099	basal RNA polymerase II transcription machinery binding	10	0.044382275
GO:0016407	acetyltransferase activity	13	0.04501654

### GO term enrichment analysis of DEG

Using the R package with data package, visualization, and integrated discovery, GO enrichment analysis was performed on 1062 DEG with different meanings. Our results revealed that 151 DEG participated in BP, 56 in CC, and 40 in MF. With respect to BP, the DEG were significantly enriched in the mRNA catabolic process, covalent chromatin modification, and histone modification. With respect to CC, they were mainly enriched in focal adhesion, cell-substrate adherens junction, and cell-substrate junction. With respect to MF, they were mainly enriched in cadherin binding, cell adhesion molecule binding, and ubiquitin-protein transferase activity (Fig. 3, Table 2).

### KEGG pathway analysis of DEG

**Figure 4:**
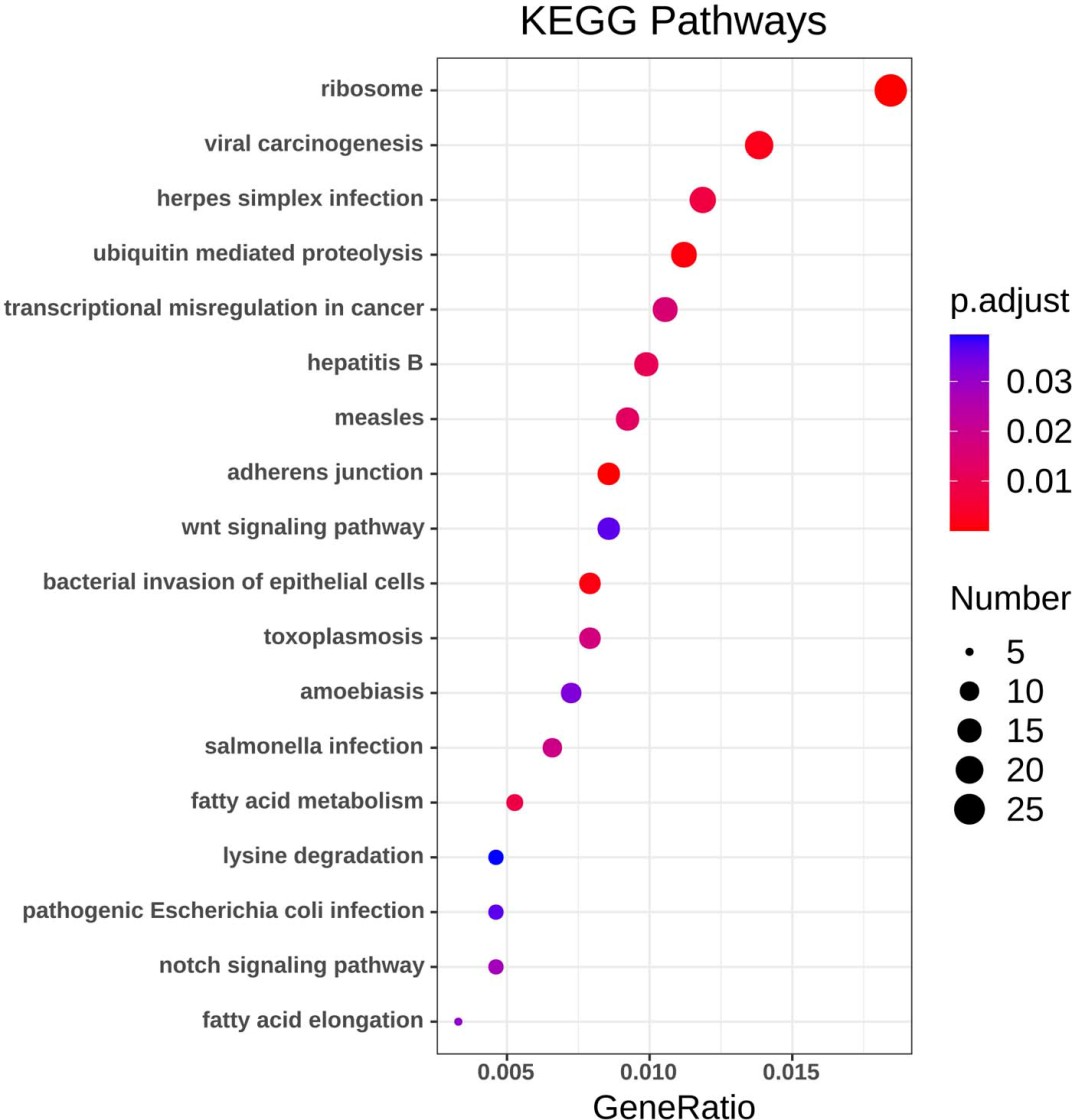
KEGG pathway analysis of DEGs. Color indicates *P* value, numbers indicate the size.

Using the R package with data package, visualization, and integrated discovery, KEGG enrichment analysis was performed on 1062 DEG with different meanings. Our results revealed that a total of 21 pathways were enriched, mainly ribosome, ubiquitin-mediated proteolysis, and cancer pathways (Fig. 4, Table 3).

### Construction of PPI network and identification of key genes

**Figure 5:**
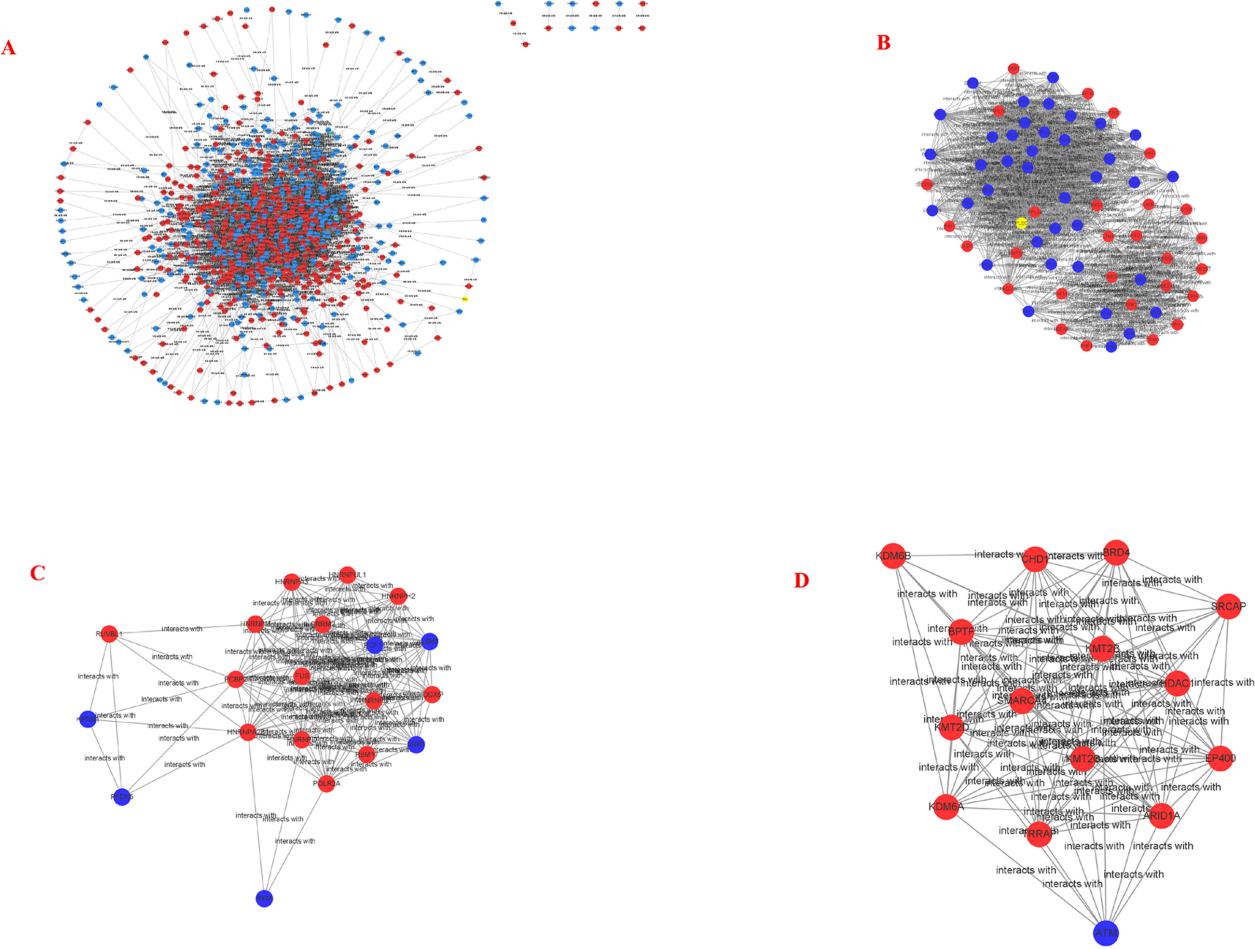
PPI network and cluster identification. (A) The interaction among 845 DEG was detected through STRING with high confidence. (B) Cluster 1 included 67 nodes and 1098 edges. (C) Cluster 2 included 20 nodes and 13 edges. (D) Cluster 3 included 15 nodes and 92 edges. The red nodes represent significantly upregulated genes, while blue nodes represent significantly downregulated genes.

STRING and Cytoscape analyses identified a total of 845 DEG participating in the PPI network, with 5,571 edges (Fig. 5A), 471 upregulation, and 374 downregulation. Through the MCODE plug-in, the first three densely interconnected clusters of the PPI network were analyzed. Cluster 1 consisted of 67 nodes and 1,098 edges. The enrichment results indicated that the genes included in Cluster 1 of the PPI were mainly enriched in the terms extracellular exosome” and “poly(A) RNA binding”. Cluster 2 was composed of 20 nodes and 13 edges. The enrichment results indicated that the genes included in Cluster 2 were mainly enriched in the terms “nuclear-transcribed mRNA catabolic process” and “acetylation”. Cluster 3 was composed of 15 nodes and 92 edges. The enrichment results indicated that the genes included in Cluster 3 were mainly enriched in the terms “transcription and chromatin regulator”, and one node was a DEG (Figs. 5B–5D; Tables 4–6). The first 30 genes in the connectivity evaluation in the PPI network were *Hub* genes (degree ≥53) (Table 7).

### Key gene expression analysis in Hp infection status

**Figure 6:**
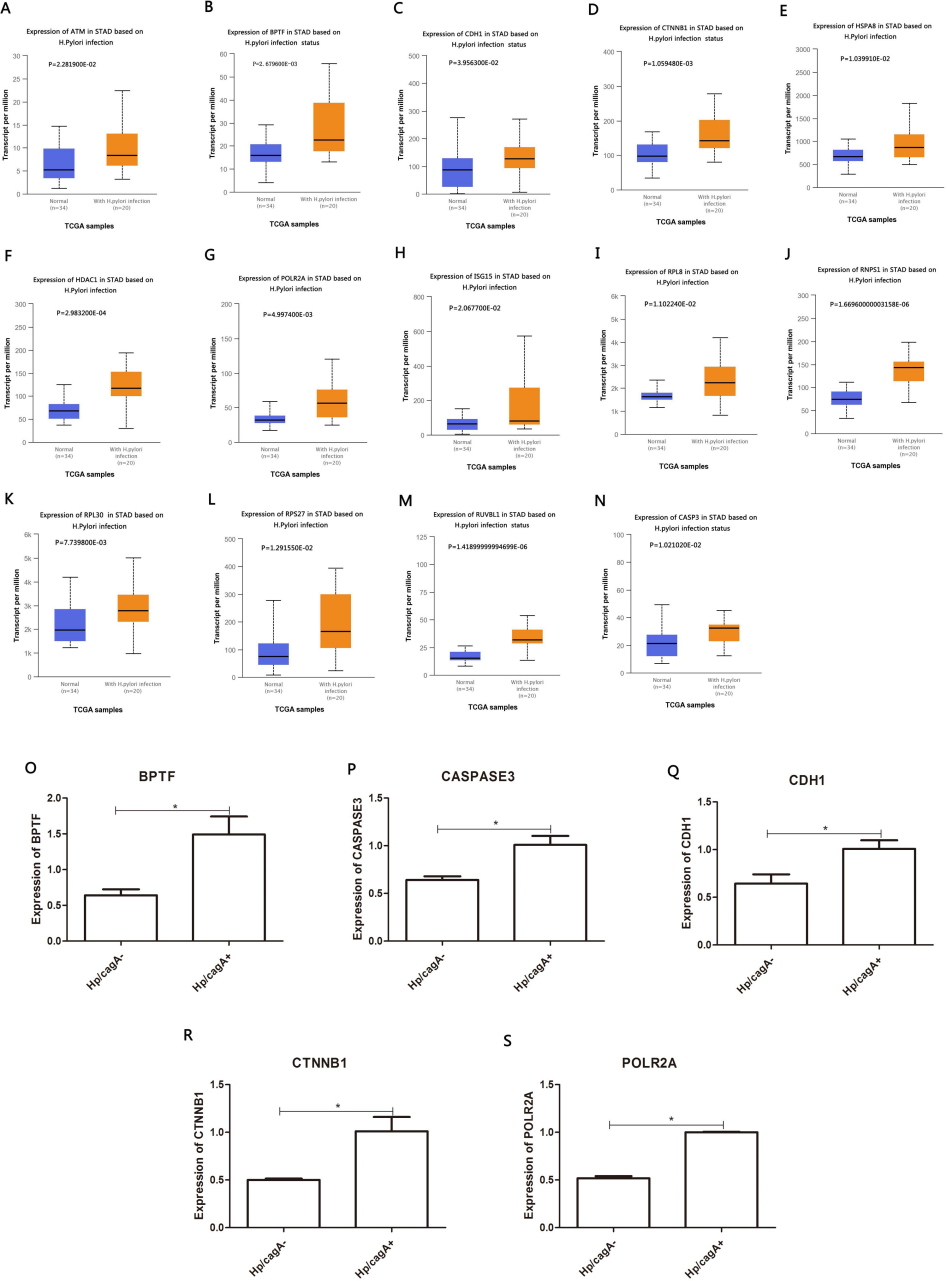
Analyze the expression and expression verification of key genes in Hp infection status according to the TCGA database. (A–N) Red color indicate expression in Hp infection status, blue color indicate expression in uninfected status. (O–S) The mRNA levels of BPTF, CASPASE3, CDH1, CTNNB1 and POLR2A by RT-qPCR. ^∗^*P* < 0.05. The *Hp*/cagA^+^ infected group compared with the *Hp*/ cagA^−^::Cm infected group*Compared between *Hp*/cagA^+^ and *Hp* △cagA group, *P* < 0.05.

The DEG identified in the PPI network (≥53) was analyzed in the TCGA database to assess the correlation with Helicobacter pylori infection. A total of 14 DEGs were highly expressed in positive Helicobacter pylori infection (*P* < 0.05) and were up-regulated by CagA, namely ATM, BPTF, CDH1, CTNNB1, HSPA8, HDAC1, POLR2A, ISG15, RPL8, RNP1, RPL30, RPS27, RUVBL1 and CASP3. RT-qPCR verification results showed that Helicobacter pylori CagA caused up-regulation of BPTF, CASP3, CDH1, CTNNB1 and POLR2A expression (*P* < 0.05) (Figs. 6A–6S).

### Survival analysis of key genes

**Figure 7:**
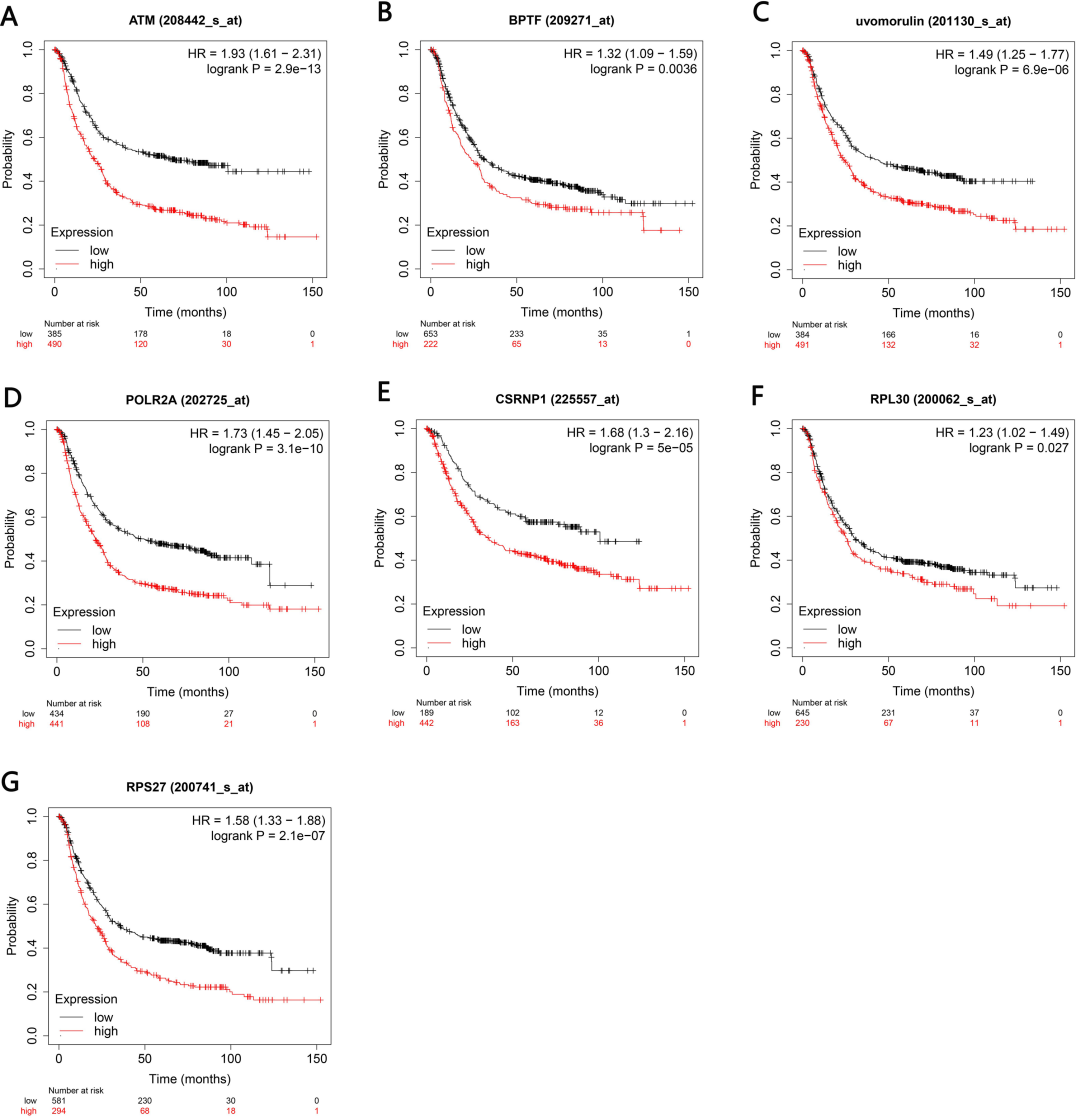
Kaplan–Meier analyses indicated the overall survival of central genesexpressed in patients with gastric cancer. (A–G) *P* < 0.05 was considered to be statistically significant. HR, hazard ratio.

The Kaplan–Meier plotter bioinformatics analysis platform was used to investigate the prognostic value of genes in 14 potential centers, including data from 875 gastric cancer patients for overall survival analysis. Our results show that under high expression (*P* < 0.05), a total of 7 genes are associated with poor prognosis of gastric cancer (*P* < 0.05), namely ATM, BPTF, CDH1, POLR2A, RNP1, BPL30 and RPS27 (Figs. 7A–7G).

## Discussion

The development of gastric cancer is an extremely complicated biological process, involving the abnormal expression of various tumor-related genes, activation of various tumor-related pathways, and inactivation of tumor suppressor genes. The causative gene is silent and inactive. In fact, evidence prove that the tumor is induced by genetic and epigenetic changes (Belinsky, 2004; Herman & Baylin, 2003; Jones & Baylin, 2002). Helicobacter pylori is closely related to gastric cancer, and Helicobacter pylori CagA is involved in multiple cellular processes related to carcinogenesis (Hatakeyama, 2017). In combination with public biological databases (such as GO and KEGG), the development of a high-throughput detection technology would facilitate systematic exploration of a list of DEG throughout the genome (Ma, Zhou & Zheng, 2020) and comb through the related BP. The application of informatics provides a good means to comprehend the mechanisms of occurrence and development of gastric cancer at the molecular level.

**Table 3:** KEGG pathway analysis of DEGs.

**Pathway**	**P-value**	**Genes**
Ribosome	2.39E−10	RPL18, RPL36A, RPL13, RPL15, RPL35, RPL36, RPL37, RPL38, RPS2, RPL30, RPS27, MRPL13, RPL31, RPL34, RPL8, RPL5, RPL11, MRPL33, RPS23, RPL35A, RPL27, RPL28, RPS7, RPL23, RPL13A, RPL21, RPL37A, UBA52
Adherens junction	1.39E−04	PARD3, PTPRF, CREBBP, CSNK2B, CTNND1, ACTN1, CDH1, CTNNA1, CTNNB1, CDC42, IGF1R, CSNK2A1, AFDN
Ubiquitin mediated proteolysis	9.53E−04	SYVN1, XIAP, UBE4A, PML, SKP1, BIRC2, STUB1, FANCL, TRIM37, FBXW7, UBE2D2, HUWE1, UBE2K, UBA3, TRIM32, NEDD4L, FBXW11
Bacterial invasion of epithelial cells	0.001284745	CDC42, PTK2, SEPT2, PIK3CB, ARPC5L, CDH1, CLTC, CTNNA1, ITGB1, CTNNB1, DNM2, SEPT9
Viral carcinogenesis	0.002245962	HIST1H4L, YWHAZ, HIST1H2BC, PIK3CB, CREBBP, UBR4, ACTN1, CDK4, PKM, CCNE2, CDC42, HDAC4, CASP3, HDAC1, GSN, GTF2A2, CREB3L2, RBPJ, HDAC8, CHD4, SYK
Pathways in cancer	0.005009934	ADCY3, FGFR2, WNT5B, XIAP, PPARG, PML, CDH1, ITGB1, TGFB1, CTNNB1, CCNE2, IGF1R, CDC42, PTK2, CASP3, RALB, FAS, RUNX1, PLCB1, CTBP2, RALBP1, PIK3CB, CREBBP, CDK4, CTNNA1, STAT1, BIRC2, ARHGEF11, HDAC1, LAMA5, PTCH1, GSTP1
Huntington’s disease	0.005587064	DNAH11, UQCRC2, COX7A2, CREBBP, PPARG, CLTC, NDUFA1, NDUFB1, NDUFB2, POLR2A, SOD2, NDUFS6, NDUFS5, NRF1, CASP3, HDAC1, CREB3L2, PLCB1, UQCRB
Non-alcoholic fatty liver disease	0.006537343	UQCRC2, COX7A2, PIK3CB, LEPR, NDUFA1, NDUFB1, TGFB1, NDUFB2, CDC42, NDUFS6, CASP3, NDUFS5, XBP1, MLXIP, FAS, UQCRB
Herpes simplex infection	0.007640717	MAVS, CREBBP, PML, CSNK2B, HCFC1, SKP1, ARNTL, STAT1, TAB2, POLR2A, TYK2, CASP3, TAF5L, CSNK2A1, TAP2, FAS, IFNGR2, IFNGR1
Fatty acid metabolism	0.008015791	CPT2, ACADM, HACD3, HACD4, FASN, HADH, HADHA, ACSBG1
Hepatitis B	0.010764364	MAVS, YWHAZ, PIK3CB, CREBBP, MAP2K4, HSPG2, CDK4, STAT1, TGFB1, STAT6, CCNE2, CASP3, CREB3L2, FAS, NFATC3
Measles	0.012473037	MAVS, PIK3CB, CSNK2B, CDK4, STAT1, TAB2, TYK2, CCNE2, CSNK2A1, FAS, IFNGR2, IFNGR1, HSPA8, ADAR
Transcriptional misregulation in cancer	0.015781479	ASPSCR1, FUS, HIST1H3J, KDM6A, KMT2A, PPARG, PML, AFF1, DDX5, HMGA2, ATM, MEN1, IGF1R, PTK2, HDAC1, RUNX1
Toxoplasmosis	0.017710762	TYK2, CASP3, XIAP, LAMA5, STAT1, BIRC2, TAB2, IFNGR2, ITGB1, TGFB1, HSPA8, IFNGR1
Salmonella infection	0.018985119	CDC42, PFN1, RILP, RAB7B, ARPC5L, DYNC2H1, KLC4, IFNGR2, DYNC1I2, IFNGR1
Notch signaling pathway	0.027873731	CTBP2, HDAC1, MAML1, DTX2, CREBBP, RBPJ, NCOR2
Fatty acid elongation	0.031084	ACOT7, HACD3, HACD4, HADH, HADHA
Amoebiasis	0.03307295	PTK2, CASP3, RAB7B, SERPINB6, LAMA5, PIK3CB, COL27A1, ACTN1, COL1A1, PLCB1, TGFB1
Wnt signaling pathway	0.036142405	NKD1, WNT5B, CTBP2, CSNK2A1, CREBBP, LRP6, CSNK2B, RUVBL1, SKP1, PLCB1, NFATC3, FBXW11, CTNNB1
Pathogenic Escherichia coli infection	0.036263427	CDC42, ARPC5L, TUBB6, CDH1, TUBB1, ITGB1, CTNNB1
Lysine degradation	0.039383478	KMT2D, KMT2A, KMT2C, SETD1A, KMT2B, HADH, HADHA

In this study, we compared 1062 genes with significant differences between the pcDNA3.1::CagA and pcDNA3.1 group via bioinformatics. Of these genes, 594 were upregulated and 468 were downregulated. Functional enrichment revealed that these genes participated in multiple signaling pathways, including the Notch signaling pathway and Wnt signaling pathway. The notch signaling pathway is a signal transduction system that repeatedly regulates cell proliferation and apoptosis. We found that the Notch signaling pathway was closely related to cell differentiation, proliferation, apoptosis, adhesion, and the transformation of epidermal cells into the mesenchyme; this pathway is essential for the normal development of most tissues (Leong & Karsan, 2006; Luo, Renault & Rando, 2005; Maillard, Fang & Pear, 2005; Zanotti & Canalis, 2016). Past studies have demonstrated that this pathway plays an important role in regulating the cell cycle as well (Bhattacharya et al., 2017; Herranz & Milán, 2008; Seidel & Kimble, 2015). In a large number of hematopoietic and solid tumors, the Notch pathway undergoes genetically alteration. The activation or inhibition of the pathway depends on the background and the activation status of other potential oncogenic pathways. There are several different patterns of abnormal regulatory pathways and their targets in cancer (Ranganathan, Weaver & Capobianco, 2011; Vasquez-Del Carpio et al., 2011; Weaver et al., 2014). These pattern include the activation and inactivation mutations, receptor/ligand overexpression, epigenetic regulation, and the effects of post-translational modifications (Wang et al., 2007). Wnt is a secreted glycoprotein that can regulate diverse biological functions (MacDonald, Tamai & He, 2009). Wnt signaling is one of the main regulators of embryonic development, tissue renewal, and regeneration in multicellular organisms (Sidrat et al., 2020; Tepekoy, Akkoyunlu & Demir, 2015). This signaling pathway controls several aspects of the development process, including cell proliferation, apoptosis, cell migration, and cell polarity during the development and maintenance of adult stem cells. Cell proliferation and apoptosis are often associated with tumor formation and development (Bordonaro, 2020; Foulquier et al., 2018; Yang et al., 2016). Inappropriate activation of the Wnt pathway is also a major factor influencing the human carcinogenesis (Martin-Orozco et al., 2019) involving 13 enriched genes.

**Table 4:** Differential genes in Cluster 1.

**Gene name**	**MCODE_Score**	**Expression**
PSMC2	22	down
RPL13	25.68403361	down
KCNC3	23	up

RPL21	25.68403361	down
RAD23B	27	up
RPL35	25.68403361	down
SRP14	26.93349754	down
RPS23	25.68403361	down
PLEC	26.45564516	up
RPL37A	25.68403361	down
RPL38	25.68403361	down
ISG15	26.93349754	down
RPL15	25.68403361	down
ERI1	23	up
UBA52	25.68403361	down
RPL11	25.68403361	down
RPL28	25.68403361	down
UBE2D2	23	up
RPL34	25.68403361	up
NEDD4L	23	up
RPL36	25.68403361	down
RNF213	23	up
RNF111	23	up
RPS2	25.68403361	down
RPL36A	25.68403361	down
SMG1	27	up
TRIM37	23	down
RPL35A	25.68403361	down
RPS27	25.68403361	down
RPL27	25.68403361	down
LAS1L	25	up
RNPS1	27	up
RPL8	25.68403361	down
RPL23	25.68403361	down
RPS7	25.68403361	up
UBA3	23	down
RPL30	25.68403361	down
RPL13A	25.68403361	down
RPL18	25.68403361	up
RPL5	25.68403361	down
RPL31	25.68403361	down
RNF6	23	down
HUWE1	23	up
GART	21.92028986	up
EIF3L	27	up
UBE2K	23	up
EXOSC8	25	down
BTF3L4	25	down
SECISBP2	26	up
TRIM32	23	up
NACA	27	down
RNF4	23	up
UBE2V1	23	up
TCEB1	23	down
TRIM4	23	up
LTN1	23	up
GAN	23	down
UBR4	23	up
UBE4A	23	up
STUB1	23	down
FBXL8	23	up
RPL37	25.94117647	down
FBXW11	23	up
MRPL13	26	down
SSR1	27	down
SKP1	23	down
FBXW7	23	up

**Table 5:** Differential genes in Cluster 2.

**Gene name**	**MCODE_Score**	**Expression**
SRCAP ARID1A ATM TRRAP KMT2B EP400 SMARCA4 HDAC1 KMT2D BPTF KDM6A CHD1 KDM6B BRD4 KMT2C	11.1 11.1 10.3956044 11.25146199 12.35 11.28947368 10.11111111 10.46769231 11.28947368 10.12681159 11.01578947 10.31578947 9.991666667 10.69264069 10.69264069	up up down up up up up up up up up up up up up

**Table 6:** Differential genes in Cluster 3.

**Gene name**	**MCODE_Score**	**Expression**
KPNB1 HNRNPUL1 DDX5 SRRT RBM17 PFDN5 RUVBL1 HNRNPA3 PCBP2 FUS HNRNPD HNRNPM HNRNPH2 HNRNPA2B1 PCF11 HNRNPU POLR2A PKM LSM7 SRRM2	18.90952381 17 17 17 17 16.9005848 15.89542484 17 17 17 17 17 17 17 17 17 17 17 17 17	down up up down up down up up up up up up up up down up up down down up

**Table 7:** Thirty hub genes in the PPI network constructed by STRING (degree ≥ 53).

**Gene symbol**	**Gene description**	**Degree**	**Express**
UBA52	ubiquitin A-52 residue	131	down
HDAC1	histone deacetylase 1	94	up
CTNNB1	catenin beta 1	89	up
POLR2A	RNA polymerase II subunit A	85	up
HSPA8	heat shock protein family A (Hsp70) member 8	79	up
CREBBP	CREB binding protein	71	up
CDH1	cadherin 1	69	up
CDC42	cell division cycle 42	69	down
SMARCA4	SWI/SNF related, matrix associated, actin dependent regulator of chromatin, subfamily a, member 4	67	up
ATM	ATM serine/threonine kinase	67	down
RPS2	ribosomal protein S2 [Homo sapiens	66	down
RUVBL1	RuvB like AAA ATPase 1	66	up
RPL11	ribosomal protein L11	63	down
RPL5	ribosomal protein L5	62	down
RPL8	ribosomal protein L8	62	down
RPL27	ribosomal protein L27	62	down
ISG15	ISG15 ubiquitin like modifier	61	down
RPL31	ribosomal protein L31	59	down
RPL15	ribosomal protein L15	59	down
RPL23	ribosomal protein L23	58	down
RPS27	ribosomal protein S27	58	down
RNPS1	RNA binding protein with serine rich doma in 1	58	up
RPL13A	ribosomal protein L13a	56	down
RPL30	ribosomal protein L30	55	down
RPL35A	ribosomal protein L30	55	down
BPTF	bromodomain PHD finger transcription	55	up
RPS7	ribosomal protein S7	54	up
RPL34	ribosomal protein L34	54	up
RPL13	ribosomal protein L13	54	down
CASP3	caspase 3	53	up

The PPI network analysis provided the interaction network with 845 genes, and the first 3 clusters with a high correlation were analyzed through the MCODE plug-in. Cluster 1 genes mainly participated in the extracellular exosome pathway, cluster 2 genes mainly participated in nuclear-transcribed mRNA catabolic processes, and cluster 3 genes were mainly involved in transcription. Some of the past studies have demonstrated that extracellular exosomes are involved in the development of tumors. The results of GO enrichment in these clusters indicate their partial relationship to tumors, suggesting that the signal molecules regulated by the Oriental strain CagA may participate in the possible molecular mechanism of tumor development.

The 30 key genes with the highest screening in the PPI network were analyzed through data, and 14 genes were highly expressed in Helicobacter pylori-positive gastric cancer patients (according to the TCGA database analysis, including ATM, BPTF, CDH1, CTNNB1, HSPA8, HDAC1, POLR2A), ISG15, RPL8, RNP1, RPL30, RPS27, RUVBL1 and CASP3). Finally, use the Kaplan–Meier plotter tool to predict the relationship between them and the poor prognosis of the patient. We have noticed that the high survival rate of these 7 genes is very low, which is related to the poor prognosis of gastric cancer, including genes ATM, BPTF, CDH1, POLR2A, RNP1, BPL30 and RPS27. The enrichment analysis of these 7 genes showed that they are related to the binding of P53, the binding of transcription factors and transcriptional regulation. After verification by RT-qPCR, the results showed that CagA of Helicobacter pylori only caused the up-regulation of 5 genes, including BPTF, CASP3, CDH1, CTNNB1 and POLR2A. Compared with survival analysis, BPTF, CDH1 and POLR2A have high gene expression and low survival rate. Past studies have reported that CDH1 gene mutations are associated with diffuse gastric cancer. This gene encodes E-cadherin, a transmembrane cadherin, and cell adhesion molecules that depend on this gene are involved in the formation of cell junctions and the maintenance of epithelial integrity (Cho et al., 2017; Figueiredo et al., 2019; Li, 2019; Van der Post et al., 2015). CDH1 is involved in mediating cell adhesion, migration, epithelial cell proliferation and cell cycle (Han et al., 2019; Pal et al., 2020). CDH1 germline mutations are associated with the encoded tumor suppressor protein E-cadherin, which is the genetic cause of hereditary diffuse gastric cancer (Van der Post et al., 2015). Among the other seven genes, BPTF is the core subunit of the nucleosome remodeling factor (NURF) complex and plays an important role in chromatin remodeling. This gene can directly activate oncogenic signals or coordinate activation with other key protein factors, thereby affecting tumor progression (Zhao et al., 2019). Human POLR2A encodes the highly conserved RPB1 protein, which is the largest of the 12 subunits of the essential RNA polymerase II (pol II) enzyme. This protein complex is responsible for the transcription of pol II encoded by all proteins. Further studies have shown that the sustained release of pol II bound to the promoter, the truncated RPB1 encoding and the shortened C-terminal domain will affect transcriptional regulation and cell cycle (Haijes et al., 2019). Based on the above analysis, BPTF, CDH1, POLR2A may be important target genes and signal molecules regulated by CagA of Helicobacter pylori and have a poor clinical prognosis. Notch and Wnt may be important signaling pathways regulated by Helicobacter pylori CagA, and play an important role in CagA regulating tumor signal molecules. Through bioinformatics analysis of the target genes and signaling pathways regulated by Helicobacter pylori CagA, and exploring the mechanism of CagA, we found that the target genes are related to the occurrence of multiple tumors in the signaling pathway. In past studies, Helicobacter pylori has a greater relationship with gastric cancer. This provides a theoretical basis for future exploration of the possible molecular mechanism of Helicobacter pylori CagA causing gastric cancer. At present, the interaction between these molecules lacks support, and experimental evidence is needed to clarify the underlying mechanism. The rise and development of the field of bioinformatics has accelerated the development of biology. Bioinformatics tools provide opportunities to deal with big data that cannot be managed manually (Wroblewski & Peek Jr, 2016)

## Conclusion

DEG of the *H. pylori* CagA plasmid group and the empty vector (negative control) group were obtained via high-throughput sequencing, followed by bioinformatics analysis using the R software, Cytoscape, and related databases. For this purpose, first, 1062 DEG with statistical significance were identified, of which 594 were upregulated and 468 were downregulated. GO enrichment and KEGG pathway analysis revealed that DEG was mainly enriched in the Wnt pathway, Notch pathway, Adhesive connection, and other pathways in cancer. To provide a theoretical basis for studying the biological processes of gastric cancer, we successfully constructed DEG PPI network, screened out 30 key genes with a relatively high degree, and further studied the network to understand the interaction among DEG. Comprehensive analysis of TCGA database, RT-qPCR and Kaplan–Meier plotter showed that Helicobacter pylori CagA can cause the up-regulation of genes BPTF, CDH1, POLR2A, and their high expression is attributable to poor clinical results. Through data analysis, these genes may be induced and regulated by Helicobacter pylori CagA. These findings enable us to understand the downstream target gene molecules and signal pathways regulated by Helicobacter pylori CagA, and provide a theoretical basis for studying the mechanism of Helicobacter pylori CagA. The target genes and signal pathways obtained in this study are related to the occurrence and development of tumors. These findings enable us to further explore and understand the basic molecular mechanism of Helicobacter pylori CagA regulating the tumorigenesis and development of target genes and signaling pathways.

##  Supplemental Information

10.7717/peerj.11203/supp-1 Supplemental Information 1 Raw data

## Additional Information and Declarations

### Data Availability

The following information was supplied regarding data availability: Raw data are available in the Supplementary Files.
